# Probiotic fermented goat milk incorporated with blackberry ( *Rubus* sp.): A novel functional food product

**DOI:** 10.1002/jsfa.70708

**Published:** 2026-05-07

**Authors:** Bibiana Bittencourt Bicca, Khadija Bezerra Massaut, Elisa dos Santos Pereira, Márcia Vizzotto Foster, Laura Martins Fonseca, Ângela Maria Fiorentini, Graciela Volz Lopes

**Affiliations:** ^1^ Department of Agro‐industrial Science and Technology Federal University of Pelotas Pelotas Brazil; ^2^ Embrapa Clima Temperado Pelotas Brazil

**Keywords:** anthocyanin, *Bifidobacterium* sp., functional foods, lactic acid bacteria, *Lactobacillus* sp.

## Abstract

**BACKGROUND:**

This study created functional fermented goat milk by adding blackberry pulp and probiotics (*Lactobacillus acidophilus* LA‐5 and *Bifidobacterium animalis* subsp. *lactis* Bb‐12). The preparation involved two variations of fermented goat milk with blackberry (*Rubus* sp.), distinguished by the absence (fermented goat milk, FGM) or inclusion of a probiotic culture (probiotic fermented goat milk, PFGM).

**RESULTS:**

Blackberry pulp was characterized by its high anthocyanin content (1614.98 mg cyanidin‐3‐glycoside per 100 g) and antioxidant activity (40 252.19 μg trolox equivalent per 100 g of dry sample). During 35 days of storage, PFGM showed higher acidity and stable, high concentrations of probiotics (>6 log CFU g^−1^), especially *Lactobacillus acidophilus*. Sensory analysis revealed high acceptability (85.5%) and purchase intention (97.5%) for PFGM.

**CONCLUSION:**

These results confirm that the developed probiotic fermented goat milk with blackberry is a stable, highly acceptable, and commercially viable novel functional food product that maintains its benefits over time. © 2026 The Author(s). *Journal of the Science of Food and Agriculture* published by John Wiley & Sons Ltd on behalf of Society of Chemical Industry.

## INTRODUCTION

Goat milk is a highly nutritious food due to its rich chemical composition, including high biological value proteins, beneficial fatty acids, and essential minerals and vitamins.^1^, ^2^ Furthermore, goat milk has lower allergenic properties than cow milk and high digestibility due to reduced fat globules and medium‐ and short‐chain fatty acids.^1^, ^3^ The supply of goat milk to consumers, in addition to its fluid form, can be done by elaborating on dairy products, including fermented milk. A novel dairy product must combine nutritional, sensory, and functional properties to meet consumer demands.

Functional foods containing probiotics provide healthy and balanced nutrition,^4^ and most of these foods come from dairy sources.^5^ Probiotics are live microorganisms that provide health benefits when consumed in adequate amounts^6^, ^7^, ^8^ and can be consumed and incorporated into fermented foods or as food supplements. *Lactobacillus acidophilus* and several *Bifidobacterium* species are probiotics available to food industries that naturally exist in the human gut.^9^ *Bifidobacterium* bacteria are the most used due to their proven safety and efficacy. *Bifidobacterium animalis* subsp. *lactis* Bb‐12 is frequently incorporated into fermented dairy products due to its stability, activity, and high viability during milk fermentation, which can enhance the rheological properties and sensory quality of the final product.^10^ *Lactobacillus acidophilus* is known for its numerous health benefits and significant use in fermenting various food products and beverages.^9^ Beyond the probiotic benefits of the mentioned bacteria, fermented milk can be incorporated with bioactive compounds to enhance its nutritional value and biological effects, and offer unique flavors.^11^, ^12^ Therefore, a functional food with appealing sensory characteristics can be developed to promote healthier eating habits and gain market inclusion.

Nondairy products can contain bioactive compounds that have positive effects on oxidative stress in the body and that can prevent cell damage.^13^ Among fruits, blackberry (*Rubus* sp.) is highly nutritious (carbohydrates, calcium, potassium, dietary fiber, and vitamins), and it is a source of phenolic compounds (mainly anthocyanins).^14^, ^15^ Anthocyanins are pigments with antioxidant, anti‐inflammatory, and antimicrobial activities and play an essential role in preventing diabetes, cancer, and neuronal and cardiovascular diseases.^14^ Consumption of blackberries has been associated with neuroprotective, hypoglycemic, hypolipidemic, antioxidant, anti‐inflammatory, anticancer, and cardioprotective effects.^16^ Recent evidence suggests a significant synergy between fruit polyphenols and the survival of probiotic bacteria in dairy matrices. Polyphenols, such as the anthocyanins found in blackberries, can act as prebiotic substrates that stimulate the growth and metabolic activity of *Lactobacillus* and *Bifidobacterium* species. Furthermore, these bioactive compounds may provide a protective buffering effect, enhancing the tolerance of probiotics to gastrointestinal stress and oxidative conditions during storage. Conversely, the metabolic activity of probiotics can biotransform complex polyphenols into simpler, more bioavailable metabolites, potentially amplifying the overall functional properties of the fermented product.^17^, ^18^

Beyond the fresh form, blackberries are processed into juices and sweets and can also be used to nutritionally enrich fermented milk, while also contributing sensory attributes like flavor and color.^11^ To our knowledge, the study presented here is the first to report the production of a fermented goat milk product incorporated with anthocyanin‐rich blackberry pulp and demonstrating probiotic effects through the addition of *Lactobacillus* and *Bifidobacterium*. Therefore, the study was designed to develop a functional fermented goat milk incorporated with blackberry pulp, *Lactobacillus acidophilus* LA‐5, and *Bifidobacterium animalis* subsp. *lactis* Bb‐12.

## MATERIALS AND METHODS

### Place of study and origin of raw material

Goat milk processed in a dairy located in Alegrete, southern Brazil, served as the raw material for fermented milk preparation. The agroindustry benefits from 900 L daily from about 150 lactating goats, mainly of the Sannen breed. The production of fermented goat milk and the analyses were carried out at the Laboratory of Processing of Products of Animal Origin and the Laboratory of Food Microbiology at the Federal University of Pelotas (UFPel, Rio Grande do Sul, Brazil).

### Freeze‐dried blackberry pulp production and characterization

Blackberry of the genus *Rubus* (cultivar Tupy) was cultivated in the experimental field of Embrapa Clima Temperado (Pelotas, Rio Grande do Sul, Brazil) without previous phytosanitary treatment. Whole fruits were frozen and lyophilized in a Liobras® Lyophilizer. Afterward, each sample was ground in a ball mill (Marconi – MA 350) and stored in an ultra‐freezer at −80 °C.^19^ The characterization of blackberry pulp was carried out through determination of pH,^20^ total titratable acidity,^20^ total soluble solids,^20^ total phenolic compounds,^21^ total anthocyanins,^22^ and antioxidant activity against 1,1‐diphenyl‐2‐picrylhydrazyl (DPPH) radical,^23^, ^24^ with adaptations.

### Production of fermented goat milk

Two fermented goat milks were produced: (i) FGM: goat milk fermented from starter cultures (*Streptococcus thermophillus* and *Lactobacillus delbrueckii* subsp. *bulgaricus*) and blackberry pulp; (ii) PFGM: goat milk fermented from a combination of starter culture (*Streptococcus thermophillus*), probiotic cultures (*Bifidobacterium animalis* subsp. *lactis* Bb‐12 and *Lactobacillus acidophilus* LA‐5), and blackberry pulp.

FGM was prepared with 1 L of pasteurized whole goat milk, 10% sucrose, 2% freeze‐dried blackberry, and 0.2% starter cultures *S. thermophilus* and *L. bulgaricus* (10 log CFU g^−1^) (YC‐380, Chr. Hansen®). First, the sucrose‐added milk was thermally treated at 95 °C for 5 min. Afterward, it was cooled to 42 °C for addition of starter cultures. Then, the formulation was incubated in a yogurt maker (FunKitchen®) for fermentation. Every 2 h, an aliquot of 10 mL of the sample was taken, and the pH and titratable acidity were checked during the entire fermentation period until reaching pH 4.6. Next, fermentation was stopped by rapidly cooling the product in an ice–water bath and storing it in a refrigerator. After cooling, the freeze‐dried blackberry was added. Finally, FGM was packaged and refrigerated at 4 °C for 35 days for the analysis.

PFGM was prepared with 1 L of pasteurized whole goat milk, 10% sucrose, 2% freeze‐dried blackberry, and 1% (11 log CFU g^−1^) combination of three strains: a starter culture (*S. thermophilus*) and two documented probiotic strains (*Bifidobacterium* Bb‐12 and *L. acidophilus* LA‐5) (ABT‐7, Probio‐Tec®, Chr. Hansen®) were used. Milk added with sucrose was thermally treated at 95 °C for 5 min. Afterward, it was cooled to 37 °C for addition of bacterial cultures. The next steps were followed according to the FGM treatment. Two replicates were performed for each treatment.

### pH and acidity analysis of FGM and PFGM

The pH value was determined using a digital pH meter (AK151, AKSO®). Titrimetric acidity was performed according to Method 947.05 of the Official Methods of Analysis of AOAC.^25^ The results were expressed as a percentage of lactic acid in 100 g of sample. Analyses were performed in duplicate during fermentation monitoring and at storage days 0, 7, 14, 21, and 35 of the fermented milk.

### Viability of starter and probiotic cultures in FGM and PFGM

For counting the FGM starter cultures (*S. thermophilus* and *L. bulgaricus*), 0.1 mL aliquots of 10^−5^, 10^−6^, and 10^−7^ dilutions were inoculated in Petri dishes containing De Man, Rogosa, and Sharpe agar (MRS, Kasvi®, India), which were incubated at 37 °C for 72 h under anaerobic conditions. The combined count of both cultures was expressed in terms of log CFU g^−1^.

The viability of starter cultures and probiotics in PFGM was determined by counting viable cells on days 0, 7, 14, 21, and 35 of storage of the fermented milk in duplicate with two repetitions.^26^ For the *L. acidophilus* count, 0.1 mL was inoculated into Petri dishes containing MRS agar supplemented with 0.02% bile salts (Sigma‐Aldrich, USA). Plates were incubated at 37 °C for 72 h under aerobic conditions. For *Bifidobacterium* Bb‐12, 0.1 mL aliquots were inoculated into Petri dishes containing LP‐MRS cysteine agar (MRS with 0.2% lithium chloride and 0.3% sodium propionate). Plates were incubated at 37 °C for 72 h under anaerobic conditions. Finally, 0.1 mL aliquots were inoculated into Petri dishes containing *Streptococcus thermophilus* (ST) agar. Plates were incubated at 37 °C for 48 h under aerobic conditions. The results were expressed in terms of log CFU g^−1^.

### Characterization of PFGM

PFGM was characterized on day 14 of refrigerated storage, evaluating fat, protein, carbohydrates, moisture, and ash. The Soxhlet method was used to determine the fat percentage using hexane as a solvent.^20^ Protein analysis was performed by determining the total nitrogen content using the Kjeldahl method and converting it into crude protein using a factor of 6.38.^20^ Carbohydrate analysis was performed by the difference between 100 and the sum of the percentages of moisture, ash, fat, and protein expressed in terms of per 100 g.^20^ The moisture percentage was determined by the oven drying method at 105 °C, and the ash percentage by the muffle furnace incineration method at 550 °C.^19^

### Microbiological analysis of PFGM

The microbiological parameters of PFGM were assessed after 14 days of refrigerated storage by analyzing mold and yeast counts, *Escherichia coli* counts, and the presence of *Salmonella* spp.^25^

For *Salmonella* research, 25 g of the product was added to 225 mL of buffered peptone water (BPW; Kasvi®), which was incubated at 37 °C for 24 h. After incubation, 0.1 mL aliquots were transferred to tubes containing 10 mL of Rappaport–Vassiliadis (RV; Kasvi®) broth and 1.0 mL aliquots to tubes containing 10 mL of tetrathionate (TT; Kasvi®) broth, which were incubated in a water bath at 42 °C for 24 h and in an incubator at 37 °C for 24 h, respectively. Aliquots of the RV and TT broths were seeded onto xylose lysine deoxycholate (Kasvi®) and Hektoen Enteric (Kasvi®) agar plates using the streak plate technique, which were incubated at 37 °C for 24 h. The plates were examined, and the characteristic colonies were subjected to biochemical confirmation. For the enumeration of *Escherichia coli*, serial decimal dilutions (up to 10^−6^) were prepared from BPW, and 0.1 mL aliquots of the dilutions were added to sterile Petri dishes, followed by the addition of TBX chromogenic agar (Kasvi®). The plates were homogenized and incubated at 44 °C for 24 h. After incubation, the typical *β*‐glucuronidase‐positive colonies were counted. For the enumeration of molds and yeasts, serial decimal dilutions (up to 10^−6^) were prepared from BPW, and 0.1 mL aliquots of the dilutions were added to the surface of potato dextrose agar (Kasvi®), acidified with 10% tartaric acid (Synth®) to pH 4.0. The inoculum was spread with a Drigalski loop, and the plates were incubated at 25 °C for 5 days, without inverting. The colonies were counted, and the result expressed in terms of CFU g^−1^.

### Sensory analysis of PFGM

Sensory analysis of PFGM was performed with 79 untrained evaluators at the Federal University of Pelotas (UFPel). Each evaluator received about 15 mL of the product in a 50 mL disposable plastic cup. Through the acceptance testing, the attributes of color, aroma, flavor, texture, and overall quality were evaluated using a 9‐point hedonic scale ranging from 1 (dislike it very much) to 9 (like it very much).^27^ At the same time, purchase intention was also assessed using a 7‐point scale, ranging from 7 (would always buy) to 1 (never buy). This study was submitted to the Ethics Committee in Research with Human Beings of the Faculty of Medicine (project number 60912222.7.0000.5317). Ethical permission was granted. All participants were informed of every detail of the scope of the research. All panelists provided informed consent prior to participation.

### Statistical analysis

Data were evaluated for normality using the Shapiro–Wilk test and homogeneity of variance using the Hartley test. Afterward, the data were submitted to analysis of variance, and, in the case of significant differences, they were compared using the *t*‐test (*P* ≤ 0.05) or the Tukey test (*P* ≤ 0.05). JAMOVI software version 2.3.13 was used to carry out the statistical analyses.

## RESULTS AND DISCUSSION

### Characterization of freeze‐dried blackberry pulp

The results of physicochemical and functional characterization of blackberry pulp are presented in Table 1. The pH value was 3.311, the acidity as a percentage of citric acid was 1.26%, and the total soluble solids were 10.2 °Brix, expected values. The total anthocyanins were 1614.98 mg per 100 g dry weight, determined through the quantification of cyanidin‐3‐glucoside. Phenolic compounds were expressed in milligrams of chlorogenic acid equivalent, and 1617.98 mg was observed per 100 g dry sample. Regarding the antioxidant activity, the trolox equivalent was analyzed, and 40 252.19 μg was observed per 100 g dry sample.

**Table 1:** Physicochemical and functional characterization of freeze‐dried blackberry pulp

Analyses	Results
pH	3.31 ± 0.01
Acidity^a^	1.26 ± 0.11
TSS^b^	10.2 ± 0.09
Anthocyanins^c^	1617.98 ± 1.31
Phenolic compounds^d^	3301.49 ± 1.47
Antioxidant activity^e^	40 252.19 ± 5.26

Mean ± standard deviation.

^a^ Expressed as % citric acid.

^b^ Total soluble solids expressed as °Brix.

^c^ Total anthocyanins expressed as milligrams of cyanidin‐3‐glucoside equivalent per 100 g dry weight.

^d^ Total phenolic compounds expressed as milligrams of chlorogenic acid equivalent per 100 mg dry sample.

^e^ Antioxidant activity expressed as μg trolox equivalent per gram of dry sample.

In the analysis of total anthocyanins, a high value was obtained compared to other studies. For the Brazilian cultivar Tupy, Chaves *et al*.^16^ found a value of 1056 mg cyanidin‐3‐glucoside per 100 g of fruit, while in the present study, anthocyanin content was 34.6% higher. Anthocyanins constitute the most prevalent subgroup within the phenolic compounds and are the primary contributors to the antioxidant potential observed in these fruits. This wide variation in anthocyanin content in blackberries is due to possible effects of climatic conditions in the cultivated region, maturation stage, species, and cultivar.^16^

Chaves *et al*.^16^ also evaluated the phenolic compounds of blackberry pulp, reporting a higher value of 11 153 mg gallic acid equivalents per 100 g. Phenolic compounds are divided into two groups related to their chemical structure: phenolic acids and flavonoids (anthocyanins and flavonols, among others).^28^ These compounds bring health benefits in the metabolism and growth of the microbiota, neutralize free radicals, regulate the activities of antioxidant enzymes, reduce oxidative stress, and increase the action of the immune system, among other benefits.^29^ Among the phenolic acids in blackberry, hydroxybenzoic and hydroxycinnamic acids stand out.^30^

The DPPH assay is a widely recognized and accepted method for assessing the radical‐scavenging capabilities of antioxidants. The antioxidant activity of the blackberry pulp studied was much higher than that found by Vizzotto and Pereira,^31^ which was 9059 μg Trolox equivalent per gram of dry sample. Several factors can influence antioxidant activity. These include the cultivar and the specific plant part, which are linked to the presence of diverse phenolic compounds. Additionally, the inherent differences in the mechanisms of action among various antioxidant assays contribute to the observed variability. Antioxidants are essential in maintaining human health, preventing and treating diseases due to their ability to reduce oxidative stress.^32^

Blackberry pulp has been applied in the formulation of spreadable pastes for dairy products^11^ and freeze‐dried milk.^33^ These applications stem from its functional properties, notably its antioxidant activity. This characteristic is highly significant in today's market for developing products with fewer artificial preservatives. Furthermore, its sensory attributes play a crucial role in consumer perception and the acceptance of new products.

Although the initial characterization of the blackberry pulp revealed a high anthocyanin content, their stability within PFGM during the 35‐day storage period is likely governed by interactions with the goat milk matrix. The high protein content and fat levels observed in the PFGM formulation may provide a protective effect against the degradation of these bioactive compounds. Specifically, milk proteins, such as caseins, can form complexes with anthocyanins, potentially shielding the flavylium cation from nucleophilic attack and oxidative stress. Furthermore, the maintenance of a relatively low pH and the formation of organic acids during fermentation by *L. acidophilus* and *Bifidobacterium* are critical factors that contribute to the stability and preservation of anthocyanin pigments. While natural degradation of polyphenols may occur over time, the high antioxidant activity originally found in the pulp likely remains functional, contributing to the product's overall status as a stable novel functional food.

### Characterization of FGM and PFGM

The pH and titratable acidity were monitored throughout the fermentation period of PFGM, which was approximately 4 h. The pH and acidity of PFGM were also evaluated during the 35 days of refrigerated storage (4 °C), with aliquots taken at days 0, 7, 14, 21, and 35. Concerning pH values, a significant difference (*P* ≤ 0.05) was observed between FGM and PFGM only on day 14 (Table 2). As for acidity, PFGM presented higher acidity on days 7, 14, 21, and 35, possibly due to *L. acidophilus* LA‐5 in the formulation.

**Table 2:** Values of pH and acidity of blackberry FGM and blackberry PFGM during refrigerated storage

Time (days)	FGM	PFGM	*P*
pH			
0	4.41 ± 0.10^a^	4.51 ± 0.08^a^	0.150
7	4.62 ± 0.09^a^	4.55 ± 0.04^a^	0.189
14	4.34 ± 0.06^a^	4.44 ± 0.00^b^	0.017
21	4.22 ± 0.20^a^	4.35 ± 0.10^a^	0.269
35	4.33 ± 0.07^a^	4.40 ± 0.02^a^	0.113
Acidity*			
0	1.04 ± 0.04^a^	1.08 ± 0.08^a^	0.339
7	1.21 ± 0.02^a^	1.78 ± 0.05^b^	<0.001
14	1.21 ± 0.14^a^	1.79 ± 0.15^b^	0.001
21	1.58 ± 0.07^a^	1.86 ± 0.08^b^	0.003
35	1.78 ± 0.12^a^	2.13 ± 0.13^b^	0.007

Mean ± standard deviation. Different lowercase letters in the same line indicate a significant difference by the *t*‐test (*P* ≤ 0.05) in the comparison between FGM and PFGM.

* Results expressed as a percentage of lactic acid in 100 g of sample.

**Figure 1:**
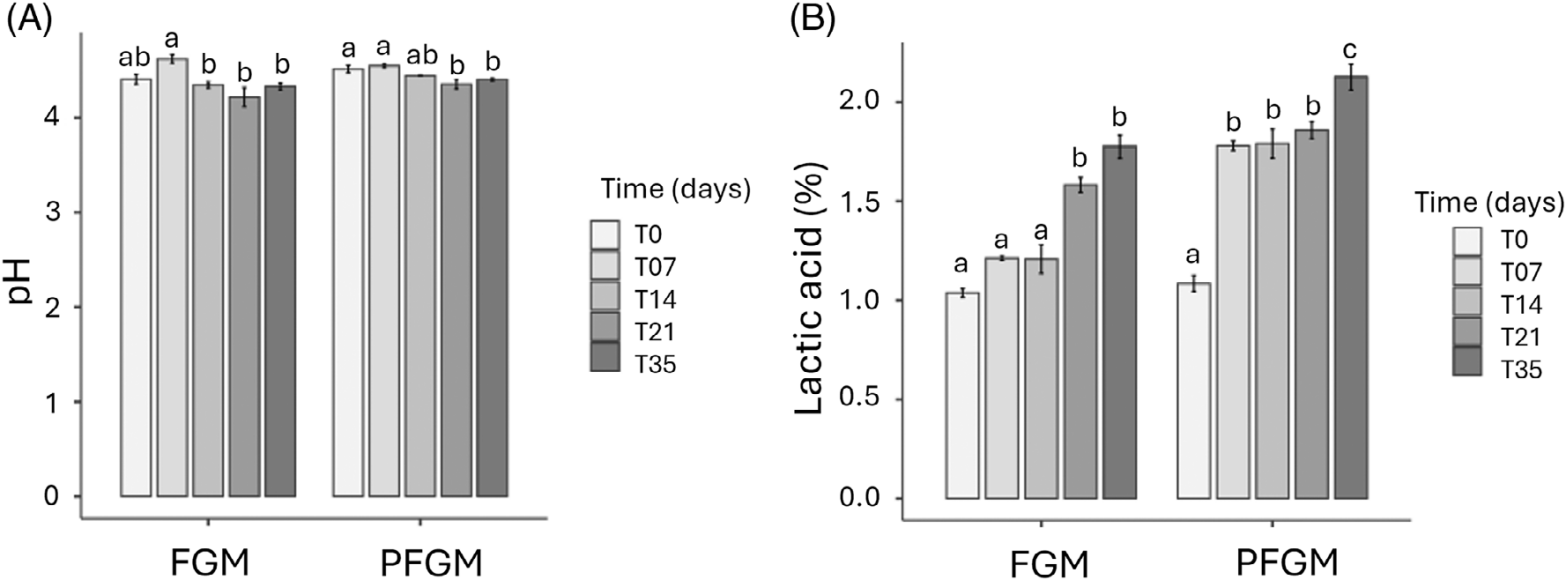
Values of pH (A) and acidity (B) in blackberry FGM and blackberry PFGM stored for 35 days under refrigeration. Different letters in the same treatment indicate a significant difference according to the Tukey test (*P* ≤ 0.05).

The influence of time on pH and acidity results is presented in Fig. 1. The pH value of FGM slightly increased on day 7, but showed no significant difference between T0 and T07 (represented by the letter ‘a’). Between T07 and T14, T21, and T35, there was a significant difference (letters ‘a’ and ‘b’). From day 14, there was no significant difference until day 35 (letter ‘b’). The pH of PFGM showed no significant change on days 0, 7, and 14 (letter ‘a’). However, a significant decrease was observed on day 21 (letter ‘b’) and remained low through day 35. Conversely, a significant increase in acidity was observed from day 21 for FGM, remaining so on day 35. The analysis of PFGM acidity revealed a significant increase at day 7. This level was sustained through days 14 and 21, after which another significant increase occurred by day 35.

The intense acidification is driven by the continued metabolic activity of *Lactobacillus acidophilus* LA‐5 and *Bifidobacterium animalis* subsp. *lactis* Bb‐12, which remains active at 4 °C. While *L. acidophilus* produces lactic acid through homofermentative metabolism, *Bifidobacterium* is responsible for acidifying the product by producing both lactic and acetic acids from residual lactose.^34^ This sustained production of organic acids accounts for the higher acidity levels in PFGM and acts as a vital preservation mechanism, maintaining a low pH that ensures product stability and safety throughout the 35‐day storage period. Thus, the fermentation process significantly impacts both the pH and acidity of milk. The characteristic decrease in pH and a corresponding increase in acidity are key indicators of successful fermentation and contribute to the unique flavor, texture, and preservation of fermented milk products.

Moreno‐Montoro *et al*.^34^ found mean pH values of 4.19 in fermented goat milk with *S. thermophilus*, *L. bulgaricus*, and *L. plantarum*. Santis *et al*.^12^ used *S. thermophilus* and *L. bulgaricus* to produce fermented goat milk and observed a pH of 4.33 at 14 days of storage, in line with what was found in this study for FGM. Regarding probiotic fermented goat milk, Machado *et al*.^35^ observed a pH value of 4.53 at 14 days of storage for probiotic fermented goat milk produced with *L. acidophilus*. Likewise, Santis *et al*.^12^ found a pH value of 4.53 at 14 days of storage in probiotic fermented goat milk with *L. lactis*, consistent with that found in the PFGM of this study, which was 4.44 at 14 days of storage.

### Viability of starter and probiotic cultures in FGM and PFGM

The viability of starter cultures was evaluated in FGM (*S. thermophilus* and *L. bulgaricus*) over time, with determinations at 0, 7, 14, 21, and 35 days of storage. Regarding the influence of time on the viability of the starter cultures, there was a reduction in initial viability up to day 35 of approximately 2 log CFU g^−1^. The initial count was 8.08 ± 0.07; 7.72 ± 0.12 at 7 days; 7.35 ± 0.11 at 14 days. However, there was a more significant reduction from day 21 (6.90 ± 0.15) to day 35 (6.17 ± 0.09) of 0.73 log CFU g^−1^. FGM conforms to the requirements of food regulations; starter cultures must remain viable at a concentration equal to or greater than 6 log CFU g^−1^ in the product until the shelf life.^36^

Yogurt fermentation occurs through the symbiotic relationship between *S. thermophilus* and *L. bulgaricus*, consequently contributing to the enhancement of the sensory, rheological, and nutritional properties of fermented milk, as well as promoting product safety due to the biopreservative function of lactic acid.^37^ Therefore, the main function of starter cultures is technological; they are responsible for the fermentation process of milk and can be combined, in a complementary way, with probiotic cultures in the production of functional foods. The influence of time on the viability of starter cultures showed that the reduction of *S. thermophilus* in PFGM was significant (*P* ≤ 0.05) on day 14 and remained at around 7.7–7.5 log CFU g^−1^. The reduction in the viability of the starter cultures in PFGM was less than 1 log CFU g^−1^ during storage of 35 days. Similar results were observed in fermented goat milk produced with starter cultures of *S. thermophilus*, *L. acidophilus*, and *B. lactis*, which maintained levels at or above 7 log CFU g^−1^ throughout the 21‐day storage period.^8^

**Figure 2:**
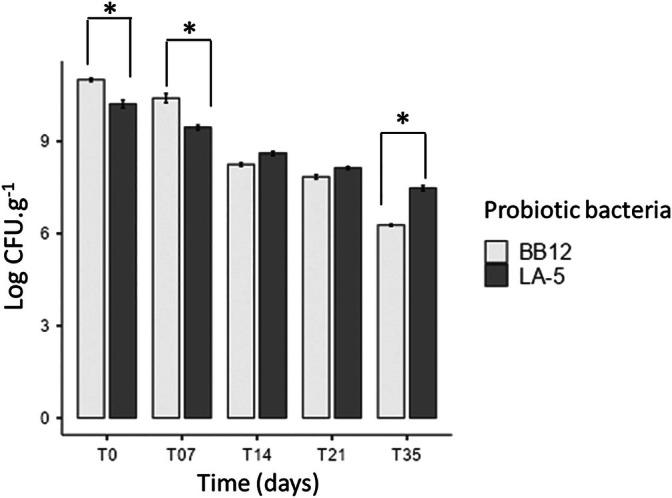
Comparison of viability of *Bifidobacterium animalis* subsp. *lactis* Bb‐12 and *Lactobacillus acidophilus* LA‐5 in blackberry PFGM stored for 35 days under refrigeration. The asterisk simultaneously indicates a significant difference according to the *t*‐test (*P* ≤ 0.05).

The viability of probiotic cultures *Bifidobacterium* Bb‐12 and *L. acidophilus* LA‐5 in PFGM was evaluated over time, with measurements at 0, 7, 14, 21, and 35 days of storage (Fig. 2). The concentrations of the isolates of *Bifidobacterium* Bb‐12 and *L. acidophilus* LA‐5 remained at high counts (>6 log CFU g^−1^) during the 35 days of storage, proving the probiotic potential of cultures used in this study and the minimum population required to provide beneficial effects on the organism.^38^ *Bifidobacterium* Bb‐12 showed a higher population on days 0 and 7 (*P* ≤ 0.05) but did not differ from *L. acidophilus* LA‐5 on days 14 and 21 (*P* > 0.05). On day 35, *L. acidophilus* LA‐5 had a higher viable cell count than *Bifidobacterium* Bb‐12 (*P* ≤ 0.05) (Fig. 2).

Several factors can affect the viability of *Bifidobacterium* Bb‐12, such as acidity, pH value, product storage temperature, and oxygen content in the package.^39^, ^40^ As *Bifidobacterium* is a strict anaerobic microorganism, in general, it tends to be more sensitive to oxygen when compared to *L. acidophilus*, demanding appropriate packaging.^41^ In addition, studies indicate that high acidity is the leading cause of the low survival of *Bifidobacterium* in fermented products with very low pH values.^41^

**Figure 3:**
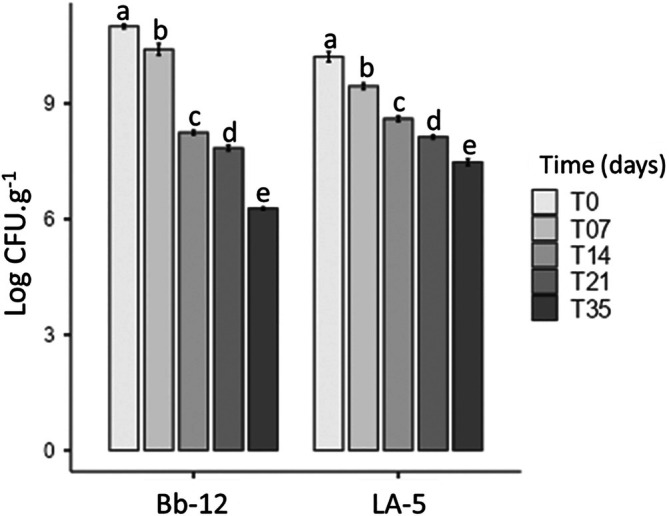
Viability over time of *Bifidobacterium animalis* subsp. *lactis* Bb‐12 and *Lactobacillus acidophilus* LA‐5 in blackberry PFGM. Different letters in the same group indicate a significant difference according to the Tukey test (*P* ≤ 0.05).

The influence of time on the viability of probiotic cultures of *Bifidobacterium* Bb‐12 and *L. acidophilus* LA‐5 can be seen in Fig. 3. Regarding *Bifidobacterium* Bb‐12, there was a more significant reduction on days 14 and 35 of 2.15 log CFU g^−1^ and 1.57 log CFU g^−1^, respectively. However, the decline was always significant (*P* ≤ 0.05). For *L. acidophilus* LA‐5, the most significant reduction in bacterial concentration was observed at 14 days of 0.85 log CFU g^−1^, with significant reductions (*P* ≤ 0.05) at all times evaluated. However, even with reduced counts, *L. acidophilus* LA‐5 showed viability above 7.4 log CFU g^−1^ at 35 days. This high viability observed for both probiotic strains throughout the 35 days of storage suggests a synergistic relationship, where the blackberry anthocyanins and phenolic compounds may have acted as protective agents against oxidative stress, while simultaneously serving as prebiotic‐like substrates that sustained microbial metabolism in the goat milk matrix.^16^, ^17^

Machado *et al*.^35^ produced fermented goat milk with *S. thermophilus* and *L. bulgaricus* and a probiotic culture of *L. acidophilus*, sweetened with honey. The *L. acidophilus* count on day 14 of storage was 7.3 log CFU mL^−1^, lower than that observed in the present study, in which the *L. acidophilus* count was 8.6 log CFU mL^−1^ on the same rated time. On the production day, PFGM showed higher values equivalent to 11 and 10.21 log CFU mL^−1^ of *Bifidobacterium* and *L. acidophilus*, respectively. Costa *et al*.^42^ observed counts of 9.49 log CFU mL^−1^ for *L. acidophilus* in probiotic fermented goat milk after fermentation. The viability of *Bifidobacterium* Bb‐12 in bovine milk was evaluated over 21 days of storage, demonstrating bacterial counts exceeding 7 log CFU g^−1^.^40^ Fermented foodstuffs represent a significant delivery system for probiotic bacteria and bioactive components, and consistent consumption of diets abundant in fermented foods has been correlated with enhanced health and greater life expectancy.^43^

### Physicochemical analyses of PFGM

The physicochemical composition of PFGM revealed 74.12% moisture content and 25.88% total solids. Specifically, it contained 4.15 ± 0.06% fat, 3.54 ± 0.05% proteins, 18.07 ± 0.18% carbohydrates, and 0.12 ± 0.01% minerals (Table 3). In comparison, Machado *et al*.^35^ reported a total solid content of 17.47%, along with 3.75% proteins, 3.01% fat, and 0.79% minerals in fermented goat milk after 14 days of storage. Similarly, Santis *et al*.^12^ analyzed fermented goat milk at 14 days, finding 88.74% moisture and 11.26% of total solids with protein and fat levels of 2.49% and 3.65%, respectively. Karagözlü *et al*.^8^ also evaluated fermented goat milk, noting fat and protein contents of 4.20% and 4.30%, respectively.

**Table 3:** Physicochemical characterization of blackberry PFGM

Analyses	Results (%)
Moisture	74.12 ± 0.06
Total solids	25.88 ± 0.04
Fat	4.15 ± 0.06
Proteins	3.54 ± 0.05
Carbohydrates	18.07 ± 0.18
Minerals	0.12 ± 0.01

Mean ± standard deviation.

### Microbiological analyses of PFGM

Microbiological analyses of *Salmonella* spp., mold and yeast counts, and *Escherichia coli* indicate that PFGM presented satisfactory microbiological parameters following the standards established by Brazilian legislation,^44^ being fit for sensory evaluation.

### Sensory analyses of PFGM

In the acceptance test, the sensory evaluation revealed that most consumers rated the assessed attributes between 7 and 9 on a 9‐point hedonic scale (1 = disliked it very much; 9 = liked it very much). The average values for each evaluated attribute correspond to *I liked it regularly/I liked it a lot/I liked it very much*, with color being the sensory attribute with the highest average (8.3), followed by texture (7.8), overall quality (7.7), aroma (7.1), and flavor (7.0). PFGM had an acceptability index of 85.5%.

The importance of color analysis cannot be overstated, given its significant influence on product acceptance and quality perception.^40^ The characteristic aroma of fermented milk products, primarily resulting from volatile compounds, plays a vital role in shaping their flavor and overall sensory appeal, making them attractive to consumers.^45^ However, the fact that goat milk is used to prepare fermented milk does not seem to have affected its aroma since 59 people out of the 79 who evaluated the product classified it as *I liked it very much* and *I liked it regularly*. It is reported that the processing of goat milk reduces the unpleasant taste, as can be confirmed in this study, as 74% of the evaluators attributed grades between *I liked it very much* and *I liked it regularly*. Regarding purchase intention, 97.5% of consumers would buy the product, indicating the potential for commercialization.

The observed sensory improvement, specifically the reduction of the ‘unpleasant taste’ often associated with goat milk, can be attributed to several biochemical changes occurring during processing. The characteristic ‘goaty’ flavor is primarily caused by the release of short‐chain free fatty acids through lipase activity. During fermentation, the metabolic activity of *S. thermophilus*, *L. acidophilus* LA‐5, and *Bifidobacterium* Bb‐12 can transform these volatile compounds into more pleasant aromatic esters and alcohols, effectively ‘masking’ or modifying the caprine profile. Furthermore, the addition of 2% freeze‐dried blackberry pulp introduces high concentrations of organic acids and sugars, which balance the sensory perception through the ‘flavor‐masking’ effect of fruit acids and the aromatic complexity of blackberry terpenes and aldehydes. This biochemical synergy likely explains why 74% of the evaluators attributed high scores to the product's flavor, supporting the potential for commercialization of this functional goat dairy product.

The high total solids content observed in PFGM likely played a fundamental role in the product's physical stability and sensory performance. This concentration is notably higher than those reported in other fermented goat milk studies. Such a robust, solid matrix, comprised of protein and fat, acts as a structural scaffold that enhances the water‐holding capacity of the protein gel. This likely prevented excessive syneresis (whey separation) during the 35 days of storage, directly contributing to the high sensory scores for texture. The presence of 2% freeze‐dried blackberry pulp further contributes to this ‘dry matter’ effect, reinforcing the viscous network and ensuring the smooth, consistent mouthfeel reported by the evaluators.

## CONCLUSION

PFGM enriched with blackberry pulp effectively maintained the viability (>6 log CFU mL^−1^) of *Bifidobacterium animalis* subsp. *lactis* Bb‐12 and *Lactobacillus acidophilus* LA‐5 throughout 35 days of refrigerated storage, suggesting potential health benefits for regular consumers. The incorporation of anthocyanin‐ and phenolic‐rich blackberry pulp not only imparted significant antioxidant properties but also positively influenced the color and overall appearance, as confirmed by sensory analysis. With a high acceptability index of 85.5% and a remarkable 97.5% purchase intent, this study underscores the promising application of innovative technologies in goat milk processing for creating appealing and functional dairy products. This aligns with the established recognition of fermented dairy foods, valued for their inherent high nutritional content derived from milk's micro‐ and macronutrients.

## FUNDING INFORMATION

This study was carried out with the support of the Coordenação de Aperfeiçoamento de Pessoal de Nível Superior – CAPES (Finance Code 001) and Fundação de Amparo à Pesquisa do Estado do Rio Grande do Sul – FAPERGS (21/2551‐0001924‐7).

## ETHICS STATEMENT

Ethical permission was granted.

## CONSENT

All panelists provided informed consent prior to participation and publication.

## CONFLICT OF INTEREST

We declare that there are no conflicts of interest.

## Data Availability

The data that support the findings of this study are available from the corresponding author upon reasonable request.
